# Relationship between super-leadership and self-directed learning ability in online nursing education: The mediating effects of self-leadership and self-efficacy perceptions

**DOI:** 10.1016/j.heliyon.2023.e17416

**Published:** 2023-06-19

**Authors:** Hye-Ja Park, Sunki Kim

**Affiliations:** College of Nursing, CHA University, 120 Haeryong-ro, Pocheon-si, Gyeonggi-do 11160, Republic of Korea

**Keywords:** Nursing students, Super-leadership, Self-leadership, Self-efficacy, Self-directed learning

## Abstract

**Background:**

Self-directed learning ability is a core competence that enables students to enhance their academic achievement, clinical competence, and professional growth in nursing education. Super-leadership—as a strategy to develop the learning process—promotes students’ self-leadership, which, in turn, enhances perceived self-efficacy perception and ultimately facilitates self-directed learning ability. Few studies have rigorously determined the influences of super-leadership, self-leadership, and academic self-efficacy perceptions on self-directed learning ability in online nursing education during the COVID-19 pandemic.

**Objectives:**

To determine the mediating effects of self-leadership and self-efficacy perceptions in the relationship between perceived super-leadership and self-directed learning ability.

**Design:**

This study was conducted using a quantitative mediation analysis design.

**Participants:**

One hundred and fifty nursing students were conveniently recruited from two nursing colleges offering the same four-year nursing degree program.

**Methods:**

Participants completed a super-leadership scale, a self-leadership scale, an academic self-efficacy scale, and a self-directed learning ability inventory. Data were analyzed with Pearson's correlations, regression analysis, Sobel's test, and Hayes's Process Macro using the bootstrap method.

**Results:**

Self-directed learning ability was correlated with perceived super-leadership (*r* = .47, *p* < .001), self-leadership (*r* = 0.69, *p* < .001), and academic self-efficacy (*r* = 0.29, *p* < .001). Super-leadership influenced self-leadership (*β* = 0.42, *p* < .001), academic self-efficacy (*β* = 0.22, *p* = .002), and self-directed learning ability (*β* = 0.36, *p* < .001). Self-leadership and academic self-efficacy mediated the relationship between super-leadership and self-directed learning ability (*β* = .42, *p* < .001; *β* = 0.35, *p* < .001) (*z* = 1.936, *p* = .026 by Sobel test).

**Conclusion:**

Learning strategies to enhance self-directed learning ability are required for successful super-leadership to boost self-leadership and academic self-efficacy perceptions among nursing students in the online, asynchronous COVID-19 educational environment. Therefore, this study's empirical evidence on integrating super-leadership with self-leadership and academic self-efficacy has practical and future research implications in terms of attaining core academic goals for nursing students.

## Highlights

• Self-directed learning ability equips nursing students with lifelong learning.
• Super-leadership develops the learning process and promotes self-leadership.
• This enhances self-efficacy and facilitates self-directed learning ability.
• Super-/self-leadership and self-efficacy influence self-directed learning ability.
• Self-leadership and academic self-efficacy have mediating effects.

## 1 Introduction

Self-directed learning ability is a core competence that equips nursing students and nurses with lifelong learning [1]. Being self-directed in terms of learning means actively identifying and taking advantage of learning opportunities [2]. Self-directed learning ability enables nursing students to enhance their academic achievement, academic self-efficacy, assertiveness, clinical competence, and professional nursing values [[3], [4], [5]].

The coronavirus disease 2019 (COVID-19) pandemic has accelerated the online pedagogical approach in nursing education over the past two years [6,7]. Self-directed learning ability continues to promote nursing students' essential competencies, such as critical thinking and problem-solving ability, within the online learning environment [8]. In contrast, academic and social pressure is building on nursing students who engage in online learning and telecommunication, since this leads to academic load, feelings of isolation and loss of personal contact with instructors and peers, increased on-screen study time, lack of standardized learning strategies, stressful evaluations and examinations, limited resources, and prolonged quarantine time [9,10]. As perceived stress with unrelenting online education negatively affects nursing students' academic and social success [11], nursing education should cultivate nursing students to become self-directed learners according to their professional needs [1,7,12]. Learners should also be more actively engaged in their own learning, as students play a significant role in their own learning within the online learning environment [7]. In addition, considering the social context where online and face-to-face classes are combined, it is necessary to devise strategies to effectively improve learners’ self-directed learning ability in the relationship between educators and learners [5,7].

Determining how nurse educators foster nursing students to be knowledgeable and competent caregivers in the online teaching-learning process has garnered much research attention [6,7]. Several previous studies have recognized the importance of educators' roles as the key success factor in higher education [6,7,12,13]. A systematic review has revealed that the continuous support and guidance provided by educators is crucial to students’ self-directed learning enhancement for lifelong learning [12]. In a previous study, the role of educators of instructional design and learning promotion had effects on learning outcomes in terms of learning competence and learning commitment [13].

**Fig. 1:**
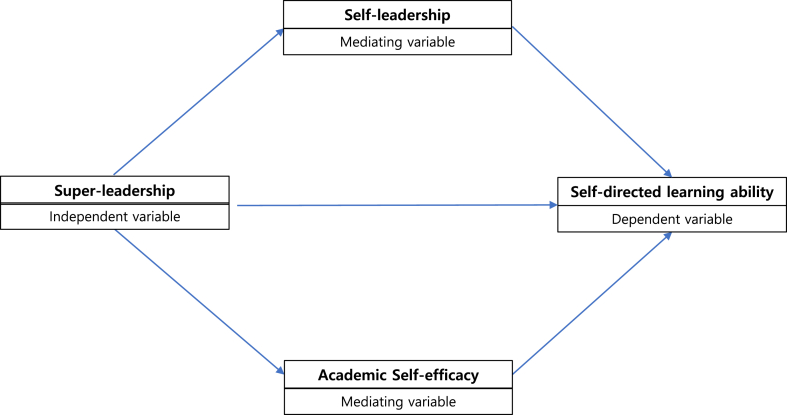
A conceptual model for self-directed learning process.

There is a theoretical model that explains the super-leadership, self-leadership, self-efficacy, and self-directed learning connection for lifelong education approaches [2]. First, super-leadership, a strategy to lead others by leading oneself, directly influences self-leadership [2,[14], [15], [16]]. The super-leadership strategies are divided into seven steps: Becoming a self-leader, Modeling self-leadership, Encouraging self-set goals, Creating positive thought patterns, Developing self-leadership through reward and constructive reprimand, Promoting self-leadership through teamwork, and Facilitating self-leadership culture [2,15,16]. Self-leadership is defined as “a comprehensive self-influence perspective that concerns leading oneself toward performance of naturally motivating tasks as well as managing oneself to do work that must be done but is not naturally motivating” [17]. The abovementioned super-leadership strategies help learners enhance their use of self-leadership [2,[14], [15], [16]]. Second, as super-leadership strategies guide educators' roles such as modeling, encouraging, guiding, and reinforcing self-direction, these approaches also influence learners' self-directed learning ability [2,16]. Third, the empowering role of super-leadership influences learners' self-efficacy perception, strengthening their self-directed learning ability in the learning process [2]. Fourth, in the self-leadership and self-efficacy theory, self-leadership focuses on self-motivation to achieve optimum performance [14,15]. It enhances one's self-directing abilities using learnable behavioral strategies (self-observation, goal-setting, self-reward, rehearsal, and self-criticism) and cognitive skills (natural rewards, self-talk, effective beliefs, assumptions, self-talk, and mental imaging pattern) [2,14]. Self-efficacy is defined as a person's belief in his or her ability to perform a certain task or behavior [18] Moreover, self-efficacy moderates one's success in the learning process and knowledge application by using strategies such as enabling successful experience, providing constructive and credible models, and providing constructive persuasive influence [2,16]. Finally, super-leadership—as a strategy to develop the learning process—promotes learners' self-leadership, which, in turn, enhances their perceived self-efficacy and ultimately facilitates self-directed learning ability [2] (Fig. 1).

Since the primary goal of super-leadership is to enable students to become self-leaders in their learning [19], effective educators encourage learners to reach their goals by strengthening their self-leadership [2,15] via self-leadership strategies [2,14]. Self-leadership and perceived self-efficacy play a major role in successful performance by influencing learners' persistent effort in self-directed learning process [14,15,18]. Previous studies have reported that nurses’ high perception of super-leadership is associated with high self-leadership and organizational commitment [20]. Self-leadership, as an essential leadership component, teaches an individual to cope with stress and facilitates self-directing learning ability among nursing students [3,11,21]. Higher self-directing or learning ability is associated with higher academic self-efficacy, self-regulating learning ability, and problem-solving ability [3]. Therefore, it will be helpful to determine the influence of super-leadership, self-leadership, and academic self-efficacy on self-directing learning ability during the current era of online teaching-learning.

However, evidence from a systematic review and meta-analysis suggests that self-directed learning ability remains insufficient and is at a moderate level among nursing students [5]; self-directed learning is not only important for enhancing the learning process, but also helps individuals in applying what is learned [2]. Nurse educators should realize the importance of self-directed learning to enhance their professional competence [5]. Self-leadership, self-efficacy, and self-directed learning are three abilities that should be enhanced through training. Mindful self-leadership training can result in better self-leadership, academic self-efficacy, and academic success while simultaneously reducing test anxiety among university students [22].

Meanwhile, few studies have rigorously determined the influences of super-leadership, self-leadership, and academic self-efficacy perceptions on self-directed learning ability in online nursing education during the COVID-19 pandemic. Examining these conceptual connections in online nursing education will be helpful for developing future nursing intervention programs. Accordingly, this study aims to determine the mediating effects of self-leadership and self-efficacy perceptions on the relationship between perceived super-leadership and self-directed learning ability. The research hypotheses according to the research objectives are as follows:

Hypothesis 1 Perceived super-leadership will be correlated with self-leadership, academic self-efficacy, and self-directed learning ability.

Hypothesis 2 Self-leadership and academic self-efficacy (mediating variables) will influence self-directed learning ability (dependent variable).

Hypothesis 3 Perceived super-leadership (independent variable) will influence self-directed learning ability (dependent variable).

Hypothesis 4 Self-leadership and academic self-efficacy will mediate the relationship between super-leadership and self-directed learning ability.

## 2 Materials and methods

### 2.1 Study design

This study employed a quantitative mediation analysis design to identify the mediating effects of self-leadership and academic self-efficacy perceptions on the relationship between super-leadership and self-directed learning ability among nursing students. Recommendations from strengthening the reporting of observational studies in epidemiology (STROBE) were used to guide this study.

### 2.2 Participants and setting

Participants were selected via convenience sampling from two universities that offer a four-year nursing degree program at Gyeonggi-do, South Korea. The eligibility criteria were as follows: the participants were nursing students between the first and fourth grades, they could respond to the online questionnaire using the Internet or a mobile device, and participation was voluntary. Participants were excluded if they had previously received extra training or coaching related to self-directed learning.

This study used G*Power 3.1.7 to estimate the required sample size [23]. According to the linear multiple regression model (two-sided test, *α* = 0.05, power 1 − *β* = 0.90, effect size f^2^ = 0.15), a sample size of 147 was required; thus, a convenience sample of 150 participants was recruited. Finally, this study used the questionnaire responses of 150 students out of 305 nursing students as an accessible population for data analyses.

### 2.3 Data collection and ethical considerations

This study was conducted in accordance with the Declaration of Helsinki and with approval from the appropriate institutional review board (IRB No: 1044308-202110-HR-083-02). Since this web-based survey was minimally risky and anonymous, and did not contain any identifying information, the IRB waived the need for informed consent (45 CFR 46.117). Information regarding IRB approval and authors’ contact information was provided on the first page of the survey.

The survey matrix was designed to be easily answered using Google Forms, which is an online survey program accessible on the Internet or a mobile device. The survey form consisted of a cover note, an informed consent form, and structured self-reported instruments. This form had been previously launched and modified by researchers. Three hundred and five nursing students registered in their nursing college's social networking sites were invited using the cover letter and hypertext link to participate in this survey, and 150 responses were finally collected. All participants were asked to complete the survey uploaded on the hypertext link after carefully reading the note on the first page, the study aims and contents, explanation regarding non-collection of personally identifiable information, confidentiality, anonymity, and their right to withdraw at any time without reprisal. The next page of the survey contained a box stating, “I agree,” which participants had to select to confirm their voluntary participation in the study. The online survey consecutively proceeded by stating that participants should respond to each question or item to minimize missing data. Each participant was provided with a small incentive of approximately $10. Data were collected from December 10–30, 2021. It took 20–30 min to fill out the questionnaire.

### 2.4 Instruments

A 96-item questionnaire was divided into five sections to collect the quantitative data.

Section 1 contained 5 items on the participants’ characteristics, including sex, grade, age, satisfaction with their major, and academic achievement, based on prior research [24,25].

Section 2 comprised 7 items to measure super-leadership using the validated Korean Self-Management Leadership Questionnaire (SMLQ) after obtaining written approval [26]; the questionnaire was originally developed and validated by Manz and Sims [17,27]. All items were measured on a 5-point scale ranging from 1 (not at all) to 5 (strongly agree); the total possible score range is 7–35, with a higher score indicating a higher level of super-leadership. Cronbach's alpha for the reliability of the SMLQ was 0.915 in the validated Korean version [26] and 0.90 in this study.

Section 3 included 35 items to evaluate self-leadership using the validated Korean Revised Self-Leadership Questionnaire (RSLQ) after obtaining written approval [28]; this questionnaire was originally developed and validated by Houghton and Neck [29]. The RSLQ included three dimensions and nine subdimensions. The behavior awareness and volition dimension consisted of five subdimensions, involving self-goal setting (5 items), self-reward (3 items), self-observation (4 items), self-cueing (2 items), and self-punishment (4 items). The task motivation dimension had one subdimension of focusing on natural rewards (5 items). The constructive cognition dimension comprised three subdimensions of visualizing successful performance (5 items), self-talk (3 items), and evaluating beliefs and assumptions (4 items). All items are measured on a 5-point Likert-type scale ranging from 1 (strongly disagree) to 5 (strongly agree); the total possible score range is 35–175, with higher scores indicating better RSLQ. Cronbach's alpha for the Korean RSLQ ranged from 0.70 to 0.80 [28]. Cronbach's alpha for the RSLQ was 0.91 and ranged from 0.71 to 0.89 in this study.

Section 4 comprised 28 items to elicit academic self-efficacy data using the validated Korean Academic Self-Efficacy Scale (ASES) after obtaining written approval [30]. The ASES consists of 3 subcategories of task difficulty preference, self-regulatory efficacy, and self-confidence. Each question is rated on a 6-point scale ranging from 1 (strongly disagree) to 6 (strongly agree); the total possible score range is 28–168, with higher scores indicating a higher level of academic self-efficacy. Cronbach's alphas for task difficulty preference (10 items), self-regulatory efficacy (10 items), and self-confidence (8 items) were 0.84, 0.76, and 0.74, respectively, in Kim and Park's study [30], and 0.71, 0.88, and 0.86, respectively, in this study.

Section 5 comprised 21 items to measure self-directed learning ability using the Korean Self-Directed Learning Ability Inventory (SDLAI) developed and validated by Bae and Lee [31]. The Korean SDLAI comprises the following three domains and seven sub-domains: management of learning process (3 items) and evaluation of learning outcome (3 items) in the cognitive domain; motivation for learning (3 items) and self-concepts (3 items) in the affective domain; and continuity of learning activity (3 items), using and management of learning resources (3 items), and making a learning environment (3 items) in the psychomotor domain. Each question is rated on a 5-point scale ranging from 1 (strongly disagree) to 5 (strongly agree); the total possible score range is 21–105, with higher scores indicating a higher level of self-directed learning ability. Cronbach's alpha for Korean SDLAI ranged from 0.68 to 0.85 [31]. Cronbach's alpha was .90 and ranged from 0.78 to 89 in this study.

### 2.5 Data analysis

The web-based data of 150 responses were coded and cleaned on Microsoft Excel spreadsheet (the Microsoft 365 version). Clean data were loaded into the statistical analysis sheet using the SPSS program (version 23; IBM Corp., Armonk, NY, USA). The participants' characteristics were analyzed using descriptive statistics such as number, percentage, mean, and standard deviation. Scores relating to super-leadership, self-leadership, academic self-efficacy, and self-directed learning ability were compared according to the participants' characteristics using the Mann–Whitney *U* test or Kruskal–Wallis test with Bonferroni's multiple comparison method, as data normality was not satisfied based on the Kolmogorov–Smirnov test. Pearson's correlation analysis was conducted to examine the relationship between super-leadership, self-leadership, academic self-efficacy, and self-directed learning ability. The mediation effect of self-leadership efficacy (mediating variable 1, M1) and academic self-efficacy (mediating variable 2, M2) on the relationship between super-leadership (independent variable, IV) and self-directed learning ability (dependent variable, DV) was analyzed using Baron and Kenny's three-step mediating regression analysis and the Sobel test. Baron and Kenny's three-step analysis [32] was conducted through the following steps: the IV's effect on M1 and M2 (step 1), IV's effect on DV (step 2), the effects of IV, M1, and M2 on DV (step 3), and a final check regarding any greater regression coefficient of the IV in step 2 than in step 3. The goodness of fit of the regression model was examined using Kolmogorov–Smirnov normality test of residuals, Durbin–Watson's range (D_L_ < Durbin–Watson's index<4-D_L_) for autocorrelation, the Koenker test for equal variance, and the variance inflation factor for multicollinearity. The direct and indirect effects of self-leadership and academic self-efficacy were verified using the Process Macro bootstrap method (number 4) [33].

## 3 Results

### 3.1 Participants’ characteristics

Regarding the participants’ characteristics, a substantial majority of them were female (n = 143, 95.3%), their mean age was 22.45 ± 4.60 years, and 74.0% were aged between 19 and 22 years. Regarding satisfaction with their nursing major, 55.4% (n = 83) reported being satisfied. Regarding academic achievement, 61.3% (n = 92) reported a moderate academic level. There were more second-grade participants than students of any other grade (n = 48, 32.0%). The mean scores of super-leadership, self-leadership, academic self-efficacy, and self-directed learning ability were 3.60 ± 0.70, 3.79 ± 0.45, 3.91 ± 0.40, and 3.86 ± 0.49, respectively (Table 1).

**Table 1:** Characteristics and means of super-leadership, self-leadership, self-efficacy perception, and self-directed learning ability among nursing students (*N* = 150).

Characteristics, Variables	Categories	Mean score range (min-max)	n (%) or M±SD
Sex	Male		7 (4.7)
	Female		143 (95.3)
School year	1st		32 (21.3)
	2nd		48 (32.0)
	3rd		30 (20.0)
	4th		40 (26.7)
Age (yr)			22.45 ± 4.60
	≤22		111 (74.0)
	≻ 22		39 (26.0)
Satisfaction in major	Unsatisfied		5 (3.3)
	Moderate		62 (41.3)
	Satisfied		83 (55.4)
Academic achievement level	Low		13 (8.7)
	Moderate		92 (61.3)
	High		45 (30.0)
Super-leadership		1-5 (1.29–5.00)	3.60 ± 0.70
Self-leadership		1-5 (2.66–5.00)	3.79 ± 0.45
Academic self-efficacy perception		1-6 (2.57–5.57)	3.91 ± 0.40
Self-directed learning ability		1-5 (2.67–5.00)	3.86 ± 0.49

M, mean; SD, standard deviation.

### 3.2 Super-leadership, self-leadership, academic self-efficacy, and self-directed learning ability according to the participants’ characteristics

The results of the Mann–Whitney *U* test or Kruskal–Wallis test with Bonferroni's multiple comparison showed that super-leadership was significantly higher among participants who were satisfied with their major (χ^2^ = 7.07, *p* = .029). Moreover, self-leadership was significantly higher among the first-grade students (χ^2^ = 9.13, *p* = .028) who were satisfied with their major (χ^2^ = 14.27, *p* = .001) and had a high academic level (χ^2^ = 14.78, *p =* .001). Academic self-efficacy was lowest among the fourth-grade students (χ^2^ = 9.51, *p* = .023) who were more than 23- year old students (z = −2.29, *p* = .023). Self-directed learning ability was highest among those who were satisfied with their major (χ^2^ = 25.55, *p* < .001) and differed among those with high, moderate, and low academic levels (χ^2^ = 26.75, *p* < .001) (Table 2).

**Table 2:** Super-leadership, self-leadership, self-efficacy, and self-directed learning ability according to characteristics (*N* = 150).

Variables		Super-leadership			Self-leadership			Academic self-efficacy			Self-directed learning ability		
Characteristics	Categories	M±SD	z or x^2^	*p*	M±SD	z or x^2^	*p*	M±SD	z or x^2^	*p*	M±SD	z or x^2^	*p*
Sex	Male	3.41 ± 0.37	−0.81	.421	3.80 ± 0.19	−0.04	.968	3.83 ± 0.52	−0.88	.380	3.77 ± 1.77	−0.64	.524
	Female	3.61 ± 0.71			3.79 ± 0.45			3.91 ± 0.39			3.87 ± 0.49		
School year	1st^a^	3.86 ± 0.69	4.52	.211	3.94 ± 0.46	9.13	.028	4.03 ± 0.44	9.51	.023	3.93 ± 0.53	5.30	.151
	2nd^b^	3.59 ± 0.84			3.85 ± 0.46		c< a	3.95 ± 0.37		d< a	3.97 ± 0.51		
	3rd^c^	3.47 ± 0.55			3.61 ± 0.48			3.90 ± 0.39			3.73 ± 0.46		
	4th^d^	3.51 ± 0.59			3.73 ± 0.33			3.75 ± 0.36			3.79 ± 0.43		
Age (yr)	≤22	3.63 ± 0.73	1.00	.323	3.81 ± 0.45	−1.02	.305	3.95 ± 0.38	−2.29	.022	3.83 ± 0.48	−1.43	.153
	≥23	3.54 ± 0.64			3.72 ± 0.44			3.77 ± 0.40			3.97 ± 0.52		
Satisfaction in major	Unsatisfied^a^	2.86 ± 0.64	7.07	.029	3.51 ± 0.61	14.27	.001	3.82 ± 0.29	0.48	.787	3.40 ± 0.35	25.55	<.001
	Moderate^b^	3.52 ± 0.62		a,b < c	3.64 ± 0.42		a,b < c	3.90 ± 0.42			3.67 ± 0.41		a,b < c
	Satisfied^c^	3.71 ± 0.73			3.91 ± 0.42			3.91 ± 0.39			4.03 ± 0.48		
Academic achievement level	Low^a^	3.46 ± 0.75	4.74	.094	3.51 ± 0.41	14.78	.001	3.76 ± 0.51	4.19	.123	3.41 ± 0.39	26.75	<.001
	Moderate^b^	3.52 ± 0.69			3.73 ± 0.42		a,b < c	3.88 ± 0.36			3.79 ± 0.43		a<b < c
	High^c^	3.81 ± 0.69			3.99 ± 0.42			4.01 ± 0.43			4.15 ± 0.48		

z-score calculated using the Mann–Whitney *U* test; **x2** calculated using the Kruskal–Wallis test; letters (a,b, c, d) presented for Bonferroni multiple comparison; M, mean; SD, standard deviation.

### 3.3 Relationship between super-leadership, self-leadership, academic self-efficacy, and self-directed learning ability

The results based on Pearson's correlation analysis showed that self-directed learning ability was significantly correlated with super-leadership (*r* = .47, *p* < .001), self-leadership (*r* = 0.69, *p* < .001), and academic self-efficacy (*r* = 0.29, *p* < .001). Additionally, super-leadership was positively correlated with self-leadership (*r* = .50, *p* < .001) and academic self-efficacy (*r* = 0.24, *p* = .003). Self-leadership was significantly correlated with academic self-efficacy (*r* = .46, *p* < .001) (Table 3). Therefore, Hypothesis 1, “Perceived super-leadership will be correlated with self-leadership, academic self-efficacy, and self-directed learning ability,” was accepted.

**Table 3:** Relationships among super-leadership, self-leadership, academic self-efficacy, and self-directed learning ability (*N* = 150).

Variables	Super-leadership	Self-leadership	Self-efficacy
	r (*p*)	r (*p*)	r (*p*)
Super-leadership	–		
Self-leadership	.50 (<.001)	–	
Self-efficacy	. 24 (.003)	.46 (<.001)	–
Self-directed learning ability	.47 (<.001)	.69 (<.001)	.29 (<.001)

M = mean; SD = standard deviation; r by Pearson's correlation analysis.

### 3.4 Mediating effects of self-leadership and academic self-efficacy on the relationship between super-leadership and self-directed learning ability

**Fig. 2:**
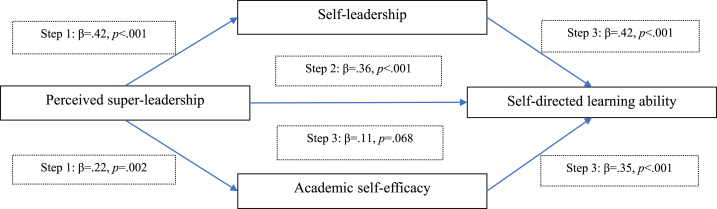
The mediating roles of learners' self-leadership and academic self-efficacy on the relationship between learners' perceived super-leadership of nursing educators and self-directed learning ability.

Baron and Kenny's mediation regression analysis revealed significant mediating effects of self-leadership (M1) and academic self-efficacy perception (M2) in the relationship between perceived super-leadership (IV) and self-directed learning ability (DV), with an appropriate goodness of fit of the regression model after adjusting for the covariates of grade, satisfaction with major, and academic achievement. In Step 1, the higher the super-leadership (IV), the greater the self-leadership (*β* = 0.42, *p* < .001) (M1) and higher the academic self-efficacy (*β* = 0.22, *p* = .002) (M2). In Step 2, the higher the super-leadership (IV), the greater the self-directed learning ability (*β* = .36, *p* < .001) (DV). In Step 3, self-leadership and academic self-efficacy completely mediated the relationship between super-leadership and self-directed learning ability (*β* = .42, *p* < .001; *β* = 0.35, *p* < .001; *z* = 1.936, *p* = .026 by Sobel test). The regression coefficient of super-leadership (IV) was larger in step 2 (*β* = 0.36) than in step 3 (*β* = 0.11) (Fig. 2). Self-leadership and academic self-efficacy explained 61% of the variance (F = 39.81, *p* < .001). Bootstrapping demonstrated two significant indirect effects between super-leadership and self-directing learning ability, with the mediating effects of self-leadership and academic self-efficacy (B = 0.18, LLCI = 0.094, ULCI = 0.260; B = 0.08, LLCI = 0.029, ULCI = 0.131) (Table 4). These results exhibited acceptable goodness of fit involving the absence of multicollinearity between the independent variables (range of variance inflation factor: 1.032–1.393), normal distribution of residuals (Kolmogorov–Smirnov's *p* > .05), absence of autocorrelation (Durbin–Watson's range: D_L_ < Durbin–Watson's index<4-D_L_), and presence of equal variance (Koenker test, *p* > .05) (Table 4).

**Table 4:** Mediating effects of self-leadership and academic self-efficacy between super-leadership and self-directed learning ability (*N* = 150).

Variables	Step 1 (X → M1)					Step1 (X → M2)					Step 2 (X → Y)					Step 3 (X, M1, M2 → Y)				
	B	*β*	SE	t	*p*	B	*β*	SE	t	*p*	B	*β*	SE	t	*p*	B	*β*	SE	t	*p*
Super-leadership (X)	1.36	.42	.22	5.95	<.001	0.67	.22	.22	3.12	.002	0.25	.36	.05	5.33	<.001	0.08	.11	.04	1.84	.068
Self-leadership (M1)																0.46	.42	.07	6.46	<.001
Self-efficacy (M2)																0.32	.35	.06	5.42	<.001
R^2^/Adj. R^2^	.338/.320						.343/.325					.379/.361					.626/.610			
F (*p*)	18.51 (<.001)						18.90 (<.001)					22.08 (<.001)					39.81 (<.001)			
Durbin–Watson's d	2.083						1.926					2.053					1.812			
dL	1.679						1.679					1.679					1.651			
4-dL	2.321						2.321					2.321					2.349			
K–S (*p*)	.044 (.200)						.058 (.200)					.064 (.200)					.060 (.200)			
Koenker test: X^2^ (*p*)	5.455 (.244)						8.165 (.086)					3.359 (.500)					3.781 (.706)			
Sobel test: z (*p*)																	1.936 (.026)			
	Effect (B)					SE	t				*p*	LLCI					ULCI			
Total effect	0.76					.14	5.33				<.001	0.476					1.038			
Direct effect	0.23					.13	1.84				.068	−0.017					0.477			
Indirect effect						BootSE						BootLLCI					BootULCI			
Total effect	0.25					.05						.164					.341			
Self-leadership	0.18					.04						.094					.260			
Self-efficacy	0.08					.03						.029					.131			

X = independent variable; M = mediating variable; Y = dependent variable; SE = standard error; LL = low level, UL = upper level; CI = confidence interval, K–S= Kolomogorov-Smirnov test for residual; Adjusted by covariates of school year (grade), satisfaction in major, academic achievement.

Therefore, Hypotheses 2 (Self-leadership and academic self-efficacy (mediating variables) will influence self-directed learning ability (dependent variable), 3 (Perceived super-leadership (independent variable) will influence self-directed learning ability (dependent variable), and 4 (Self-leadership and academic self-efficacy will mediate the relationship between super-leadership and self-directed learning ability) were supported in this study.

## 4 Discussion

In light of specific hypotheses on the self-directed learning process, this study identified the positive associations between super-leadership, self-leadership, academic self-efficacy, and self-directed learning ability among nursing students [2]. Furthermore, our findings showed that self-directed learning ability was facilitated by educators’ super-leadership and was enhanced by the mediating roles of self-leadership and academic self-efficacy among nursing students in the unrelenting online education environment amid the COVID-19 pandemic.

While nursing professionals require a more knowledgeable and practical education [1], there was no option but to shift entirely to the online teaching-learning mode without sufficient pedagogical preparation during the COVID-19 pandemic [34]. Before the advent of COVID-19, most nursing students had been performing various intellectual and experiential self-directed activities for promoting their learning by means of visual and audio sensory perceptions, knowledge construction, problem-centered orientation, and interaction with others [1]. Irrespective of changes being implemented or not, it is imperative that the nurse faculty systematically research student outcomes based on modifications made during the pandemic [6]. In accordance with systematic evidence, self-directed learning ability was developed through interactions with environmental (study programs, study years, and teaching-learning strategies) and personal factors (personal characteristics: age and gender; learning attributes: problem-solving ability, self-efficacy, learning attitude and learning interest) [12].

Super-leadership, recognized as a teaching-learning strategy, is an essential factor for facilitating learners' self-leadership, self-efficacy, and self-directed learning ability [2,[14], [15], [16]]. Nursing educators should become role models by encouraging students to set goals, creating a positive thinking style, rewarding and constructively rebuking themselves, and promoting teamwork and promoting a culture of self-leadership [2,14,15]. Compared to a previous report [26], the present study showed a slightly higher perceived super-leadership based on the measuring scale. Self-leadership, academic self-efficacy, and self-directed learning ability were also slightly higher on each measuring scale compared to the previous studies; these values were not far from those obtained through traditional didactic education [3,5]. Our results indicate that e-learning helped in providing inclusive education that set aside individuals' worries owing to the time of crisis [34]; nursing students' perseverance and inquisitiveness regarding their learning were maintained [1]. Nurse educators’ synchronous tele-lecturing and asynchronous video lectures using the technology and learning management system may facilitate teaching-learning activities in a new online learning environment [5,34]. However, great efforts are required to enhance the super-leadership, self-leadership, self-efficacy, and self-directed learning ability of nursing students, which remained at a moderate level in this study.

Regarding the learners' personal factors, highest satisfaction with their major was significant in increasing their super-leadership, self-leadership, and self-directed learning ability. Students with the highest academic achievement had the highest self-leadership and self-directed learning ability scores. This may be explained by students with higher academic success being highly motivated to develop and sustain their education as self-directed learners; they may do this by being open to learning, monitoring their learning, self-regulating, and continuing their engagement for learning [25]. In contrast, as some students progressed in their studies, their academic burdens became aggravated with distance online learning due to the huge daily workload, and compression of the curriculum into a shorter time [9,13]. Exceptionally, the third-grade students had the lowest mean scores in self-leadership, while fourth-grade students had lower self-efficacy score than did first-grade students in this study. Since the third- and fourth-grade students enrolled in more accelerated learning courses along with clinical courses, students experienced academic burden in clinical practice nursing with respect to dissatisfaction with clinical courses during the COVID-19 pandemic [9,13]. The third- and fourth-grade students could return to clinical practice after passing weekly polymerase chain reaction tests for COVID-19, according to the latest health policy established in May 2023. Since the safety of the patients, students, and faculty was prioritized [34], clinical practice was not fully open to nursing students and was replaced with an alternative intermittent virtual video simulation practice under educators' limited guidance and coordination. As the COVID-19 pandemic created a lot of chaos and tension in the clinical practical learning environment, students spent their whole day in online classes, where they watched videos/listened to audio and completed assignments [9]. The teaching-learning environment may interfere with the development of competencies in the learning process [12]; hence, it is suggested that instructional environments be designed in a way to develop more effective learning strategies and create more space for activities in practical settings [25,35]. Meanwhile, in the ongoing COVID-19 pandemic, nursing education has evolved with the modernization of teaching by using the tools of Information and information and communications technologies such as simulations, virtual learning objects, hypermedia, web platforms, videos, and cellphone applications in distance education, despite the issues to develop educators’ abilities and competencies in the development and use of these technologies [34,36].

With respect to the relationship between super-leadership, self-leadership, academic self-efficacy perceptions, and self-directed learning ability, the latter is significantly intertwined with the others, as proposed in the conceptual model for facilitating self-directed learning [2]. The results may be explained by systematic evidence showing that self-directed learning interacts with teaching-learning strategies and personal learning attributes of problem-solving ability, self-efficacy, learning attitude, and learning interest [12]. Educators’ competencies to support and guide students enable them to be self-directed learners and undergo lifelong learning during study [9,12]. The noticeable differences in our study involved the school year (age), satisfaction with major, and academic achievement. Since such factors have environmental and personal influences on self-directed learning ability, they must be considered as confounding variables that could affect the relationship between other variables [12].

Regarding the specific hypotheses, this study determined that learners' self-leadership and academic self-efficacy played a significant mediating role in enhancing the self-directed learning ability associated with learners' perceived super-leadership of their educators. First, students' perception regarding the impact of educators' super-leadership on self-leadership explained 32.0% of the variance in our study, and self-efficacy explained 32.5% of the variance. Second, super-leadership explained 36.1% of the variance in predicting self-directed learning ability. Therefore, pursuing educators' professional development is greatly important to help learners facilitate their autonomy and responsibilities [2,37]. Furthermore, an inclusive framework should be designed for educators’ role by incorporating a developmental continuum, mapping roles to appropriate levels of expertise in teaching-learning strategies, and assigning progression indicators in online nursing or contact education [37]. Finally, the mediating effects of self-leadership and self-efficacy explained 61.1% of the variance in the relationship between super-leadership and self-directed learning ability. Self-leadership could be a powerful motive for adapting and improving competency and work satisfaction [38]. Academic self-efficacy could be a driving force for finding solutions to complex problems [3]. Hwang and Oh [3] revealed that academic self-efficacy is influenced by self-directed learning ability, while Song et al. [8] emphasized the mediating role of self-directed learning ability in the relationship between critical thinking and problem-solving ability. Thus, self-directed learning ability may have meaningful bidirectional links among various learning competencies involving academic self-efficacy, critical thinking, problem-solving ability, and self-regulated learning. Given its positive role in nursing education, it is essential that nursing professors impart self-directed learning skills to students via training courses in online education [5,8,12,22].

This study's limitations are as follows: First, the results cannot be generalized to other settings because the participants were recruited from two nursing colleges in South Korea. Future studies with more representative and larger number of participants from various institutions are required. Second, because the data underwent cross-sectional analysis, further longitudinal studies will be needed to identify the strengths and weaknesses in online learning to reinforce self-directed learning ability.

Further research is needed to prove the effect of improving self-directed learning ability by planning classes using strategies based on super-leadership, self-leadership, and academic self-efficacy; and adapting them for teaching and learning.

## 5 Conclusions

Self-directed learning ability is a core competence that equips nursing students with lifelong learning. This study determined the mediating effects of self-leadership and academic self-efficacy perceptions on the relationship between super-leadership and self-directed learning ability in online education during the COVID-19 pandemic. The results revealed that self-directed learning ability was associated with perceived super-leadership, self-leadership, and academic self-efficacy. Super-leadership influenced self-leadership, academic self-efficacy, and self-directed learning ability. Self-leadership and academic self-efficacy had a significant mediating effect on the relationship between super-leadership and self-directed learning ability in online learning. Based on these findings, it will be crucial to develop and implement appropriate self-directed learning programs in accordance with super-leadership, self-leadership, and academic self-efficacy strategies for nursing students during the online learning process. This empirical evidence on integrating super-leadership with self-leadership and academic self-efficacy has practical and future research implications in terms of attaining core academic goals for nursing students.

## Production notes

### Author contribution statement

Hye-Ja Park, Sun ki Kim: Conceived and designed the experiments; Performed the experiments; Analyzed and interpreted the data; Contributed reagents, materials, analysis tools or data; Wrote the paper.

### Data availability statement

Data will be made available on request.

### Funding statement

This work was supported by the industry academic cooperation foundation fund, CHA University Grant (CHA-202200320001).

## Declaration of competing interest

The authors declare that they have no known competing financial interests or personal relationships that could have appeared to influence the work reported in this paper.
